# Anatomical Pitfall in Calot’s Triangle: Safe Cholecystectomy in the Presence of Moynihan’s Hump

**DOI:** 10.7759/cureus.95889

**Published:** 2025-11-01

**Authors:** Aung Chan Thar, Khadheeja Abdulla, Azwar Anjum

**Affiliations:** 1 Department of General Surgery, Kulhudhuffushi Regional Hospital, Kulhudhuffushi, MDV

**Keywords:** anatomical variation, calot's triangle, critical view of safety (cvs), laparoscopic cholecystectomy, moynihan's hump, right hepatic artery (rha)

## Abstract

Laparoscopic cholecystectomy is one of the most frequently performed surgical procedures, with a low overall complication rate; however, anatomical variations within Calot’s triangle can pose significant risks if not properly recognized. Among these, Moynihan’s hump - a tortuous right hepatic artery (RHA) passing unusually close to the gallbladder and cystic duct - represents a rare but important vascular anomaly. We report the case of a 25-year-old woman with symptomatic cholelithiasis who underwent laparoscopic cholecystectomy. Intraoperatively, a tortuous RHA, forming a Moynihan’s hump with a short cystic artery arising from it, was encountered. Careful dissection above the sulcus of Rouviere allowed the attainment of the critical view of safety, ensuring safe ligation and division of the cystic artery and duct. The patient’s postoperative recovery was uneventful. Moynihan’s hump is a potential anatomical pitfall that may lead to inadvertent vascular or biliary injury if unrecognized. Awareness of such variations, meticulous dissection, and strict adherence to the critical view of safety are essential to preventing operative complications and ensuring safe outcomes in laparoscopic cholecystectomy.

## Introduction

The field of laparoscopic surgery has seen major advancements and the introduction of newer techniques over the past decades. Regardless of the various modalities and technologies available, a thorough understanding of anatomy and its numerous variations remains essential to ensure safe and successful surgery. Laparoscopic cholecystectomy is one of the most commonly performed elective procedures by general surgeons, with a reported complication rate of only 0.5%-6%, of which major vessel injury accounts for approximately 0.04%-1% [1]. Anatomical variations in the biliary tree and surrounding structures are well documented, among which variations in the origin and course of the right hepatic artery (RHA) are particularly noteworthy.

The celiac trunk gives rise to the common hepatic artery, which divides into the gastroduodenal artery and hepatic artery proper. The right and left hepatic arteries then branch from the hepatic artery proper. Ligation of the cystic artery, which commonly arises from the RHA, is a critical step in gallbladder and hepatobiliary surgery. In some cases, the RHA courses very close to the gallbladder, cystic duct, and common hepatic duct (CHD) [2]. Although variations in the origin and course of the RHA are well documented, a tortuous, “hump-like” RHA is rare. This configuration forms the so-called “Caterpillar hump” or “Moynihan’s hump” and accounts for approximately 7% of all RHA variations [3]. This anatomical configuration is a critical surgical pitfall that, if unrecognized, may lead to catastrophic outcomes. Thus, a precise understanding of hepatobiliary anatomy and its variations is essential for performing a safe cholecystectomy.

## Case presentation

**Figure 1:**
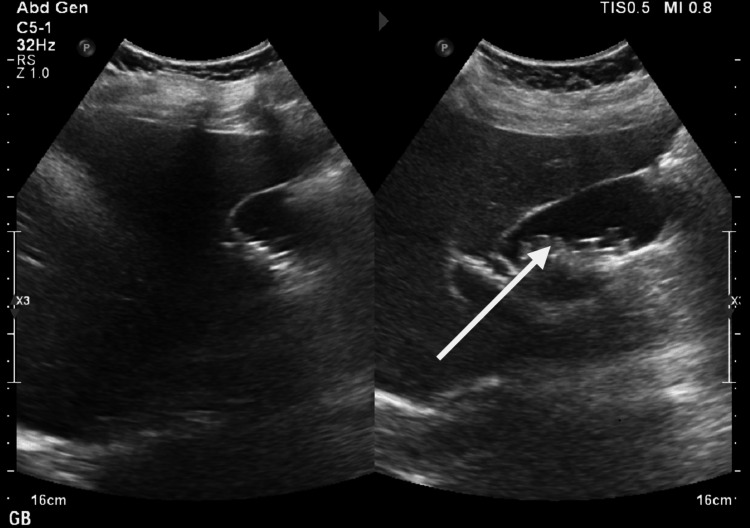
Ultrasound showing multiple gall stones

A 25-year-old female with no significant past medical history presented with dyspepsia and intermittent right hypochondrial pain for two months. The pain was often triggered by fatty meals, but she had not sought medical care before. There was no history of fever or jaundice. The examination findings were unremarkable. Baseline investigations, including complete blood count, liver function tests, renal function tests, and electrolytes, were within normal limits. Ultrasonography of the whole abdomen revealed cholelithiasis with multiple calculi in the gallbladder, the largest measuring 7 × 8 mm (Figure 1). After counselling the patient regarding risks and complications and obtaining informed consent, a laparoscopic cholecystectomy was planned.

**Figure 2:**
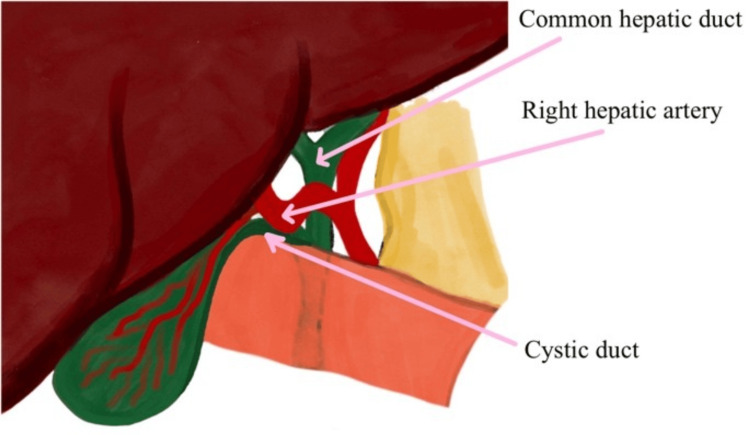
Illustration showing the tortuous hump-like right hepatic artery seen within the boundaries of Calot’s triangle Image credit: Khadheeja Abdulla

During the procedure, the gallbladder appeared distended and contained multiple calculi, one of which was located in Hartmann’s pouch. An anomalous artery was observed traversing Calot’s triangle. Initially, the lateral and medial peritoneal surfaces corresponding to the cystic plate were carefully dissected above the line of the sulcus of Rouviere to achieve a critical view of safety. At this stage, the anatomical variant became evident: the RHA formed a loop within Calot’s triangle, running very close to the cystic duct, consistent with Moynihan’s hump (Figure 2).

**Figure 3:**
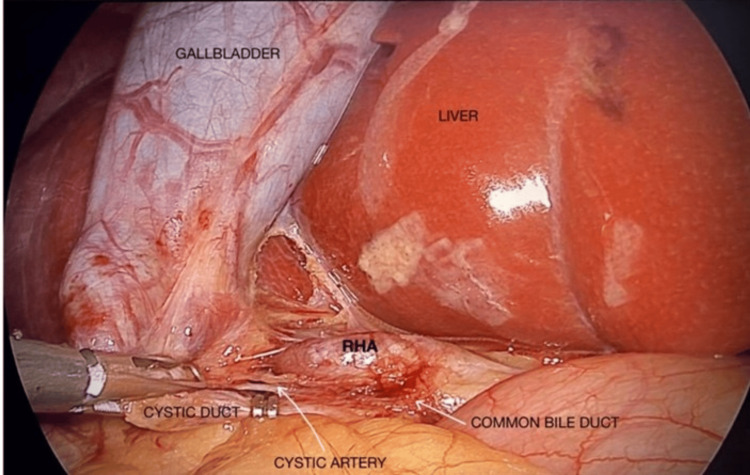
Intraoperative image showing the right hepatic artery(RHA) in Calot's triangle in the form of Moynihan's hump RHA sits anterior to the cystic duct.

According to the Nagpur classification [2], the finding corresponded to a supracystic anterior type of caterpillar hump of the RHA. Careful dissection subsequently revealed a small, short cystic artery originating from the tortuous RHA. The critical view of safety was achieved by clearly identifying the tortuous RHA, the short cystic artery, and the cystic duct (Figure 3). The cystic artery was divided using a Surginova device, and the cystic duct was clipped and divided. The gallbladder was then dissected from the liver bed. The postoperative course was uneventful, and the patient was discharged on the second postoperative day.

## Discussion

Since the introduction of open and later laparoscopic cholecystectomy, surgeons have placed particular emphasis on the anatomy of the hepatobiliary triangle to ensure safe dissection. Anatomical variations in this region are reported in 20%-50% of cases [3], and a thorough understanding of these variations is essential, as the exact morphology can only be established through meticulous dissection during surgery. Vascular injuries in this area remain one of the most significant intraoperative complications and may sometimes necessitate conversion from laparoscopic to open cholecystectomy.

The incidence of vascular injury leading to conversion from laparoscopic to open surgery ranges from 0% to 1.9%, with an associated mortality rate of up to 0.02%. [4,5]. One notable variation is the “caterpillar hump” or Moynihan’s hump of the RHA, reported in 1.35-13.3% of cases [6]. The RHA may pass either anterior or posterior to the common hepatic duct [7], and cadaveric studies have shown that the posterior course is more common, occurring in about 60% of cases [8]. In our case, the RHA formed a single loop anterior to the common hepatic duct, with a short cystic artery arising from its distal segment. Complete ligation of the RHA can result in ischemic necrosis of the right hepatic lobe, whereas partial injury may lead to hepatic artery pseudoaneurysm [9]. Additionally, uncontrolled hemorrhage can obscure the operative field, increasing the risk of bile duct injury.

Intra-operative cholangiography (IOC) during laparoscopic cholecystectomy is a valuable adjunct that provides real-time visualization of the biliary tree, aiding in the identification of the cystic duct, common hepatic duct, and anatomical variations [10]. It is particularly useful in cases with inflammation, adhesions, or unusual vascular anatomy, as it helps reduce the risk of inadvertent bile duct injury. However, IOC still requires careful dissection in potentially hazardous areas and does not substitute for meticulous surgical technique or comprehensive knowledge of hepatobiliary anatomy.

Intraoperative ultrasonography (IOUS) serves as another useful adjunct during laparoscopic cholecystectomy, offering real-time imaging of the biliary anatomy without the need for contrast injection. According to Yamashita et al., IOUS, particularly when combined with color Doppler imaging, allows clear visualization of the common bile duct, hepatic ducts, and cystic duct, as well as adjacent vascular structures [11]. This technique can accurately identify anatomical variations, biliary stones, and vascular anomalies, providing valuable information during dissection. It is especially advantageous when IOC is unavailable or contraindicated, as it avoids the potential complications associated with contrast use and radiation exposure. Thus, IOUS with color Doppler offers a safe and effective alternative for delineating both biliary and vascular anatomy during surgery.

In the present case, safe dissection was achieved through meticulous identification of anatomical landmarks and careful recognition of an unusual vascular configuration within Calot’s triangle, accomplished without the use of IOC or IOUS.

Adherence to the “critical view of safety” (CVS) principle remains indispensable. By ensuring that only two structures - the cystic duct and cystic artery - are conclusively identified before division [12], CVS provides a structured safeguard against misidentification, even in the presence of rare vascular variations. This case reinforces the relevance of CVS and demonstrates how precise anatomical understanding can compensate for unexpected intraoperative findings.

This case contributes to existing knowledge by highlighting the anatomical pitfall posed by Moynihan’s hump and emphasizing the importance of vigilance within Calot’s triangle. Maintaining a clear operative field, proceeding with controlled dissection, and respecting anatomical variability are the cornerstones of safe cholecystectomy.

Ultimately, this case serves as a reminder that safe cholecystectomy depends not on technological adjuncts alone, but on the surgeon’s anatomical knowledge, vigilance, and disciplined adherence to the critical view of safety.

## Conclusions

Anatomical variations within Calot’s triangle, such as Moynihan’s hump, continue to pose significant challenges during cholecystectomy. Careful and meticulous dissection, combined with strict adherence to the principles of the critical view of safety, remains the cornerstone for preventing such complications and ensuring safe surgical practice. Surgeons should maintain a high index of suspicion for these anomalies and proactively anticipate their presence, as timely recognition not only reduces the risk of inadvertent injury but also prevents the need for conversion to open surgery.
